# Supplementary figures and images for: Deciphering the genetic basis of grain iron and zinc content in wheat under heat and drought stress using GWAS
Source: PLoS One. 2025 Aug 14;20(8):e0329578. doi: 10.1371/journal.pone.0329578 (PMC12440253; doi:10.1371/journal.pone.0329578)

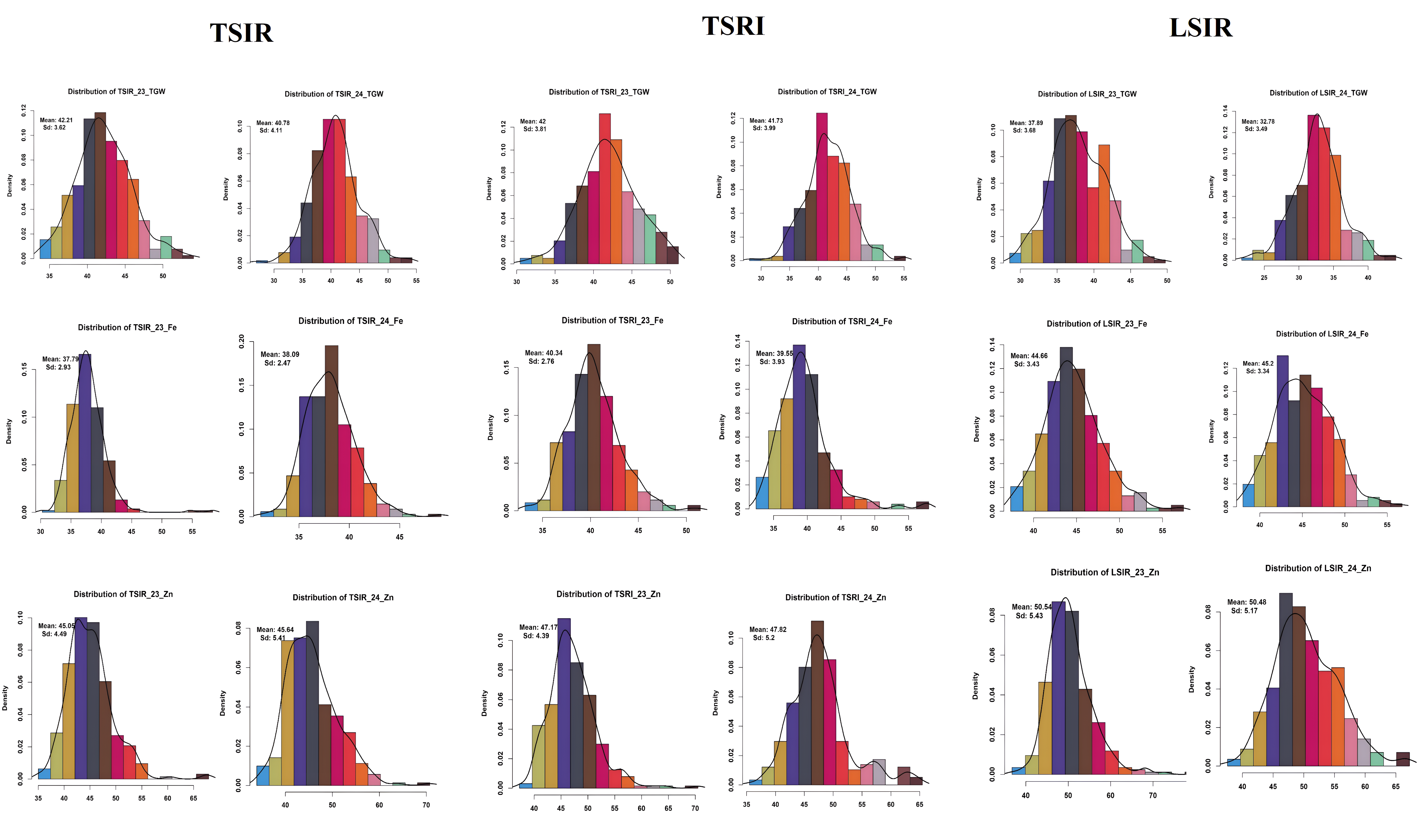

Supplement: S1 Fig — (TIFF) [file pone.0329578.s001.tiff]

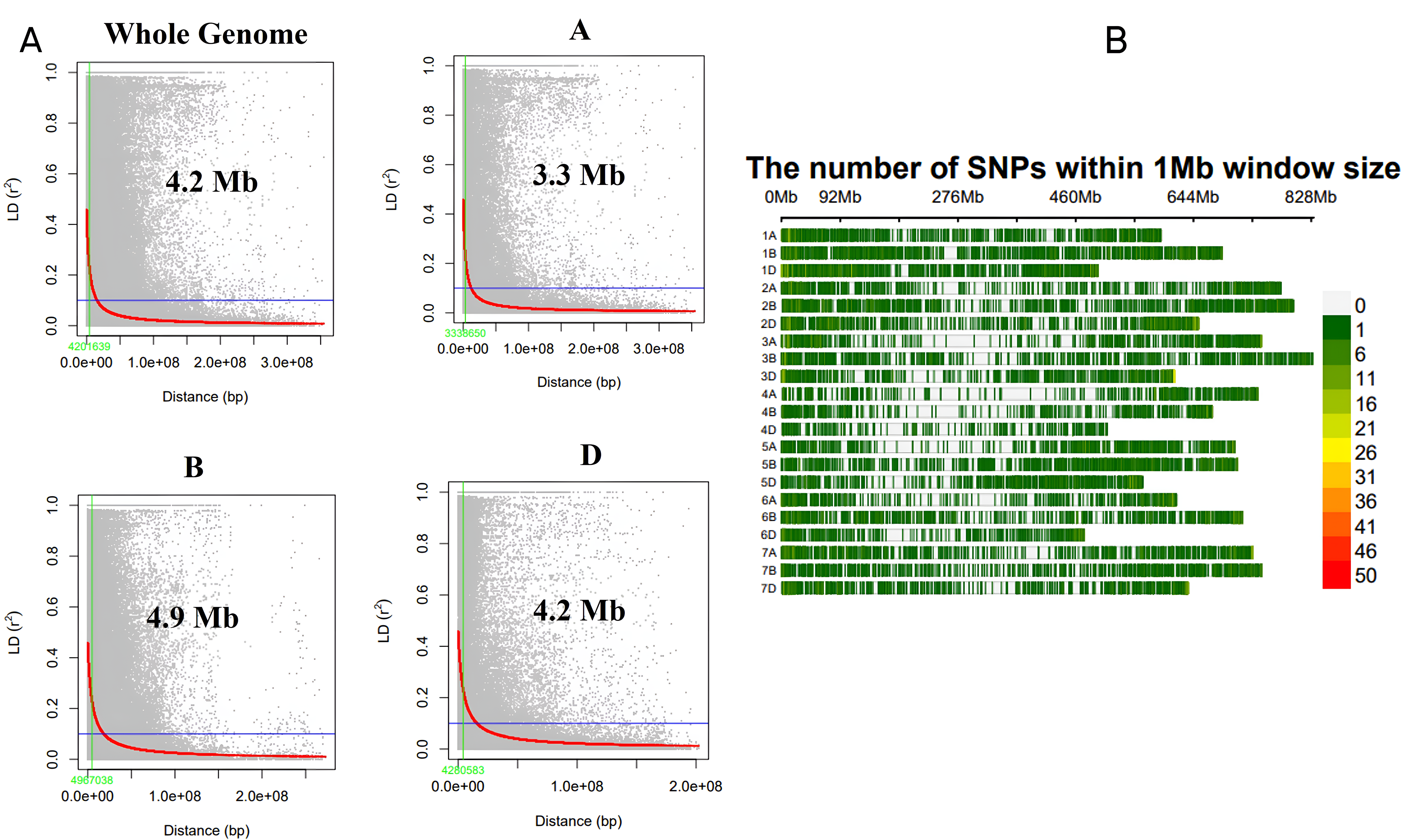

Supplement: S2 Fig — (TIFF) [file pone.0329578.s002.tiff]

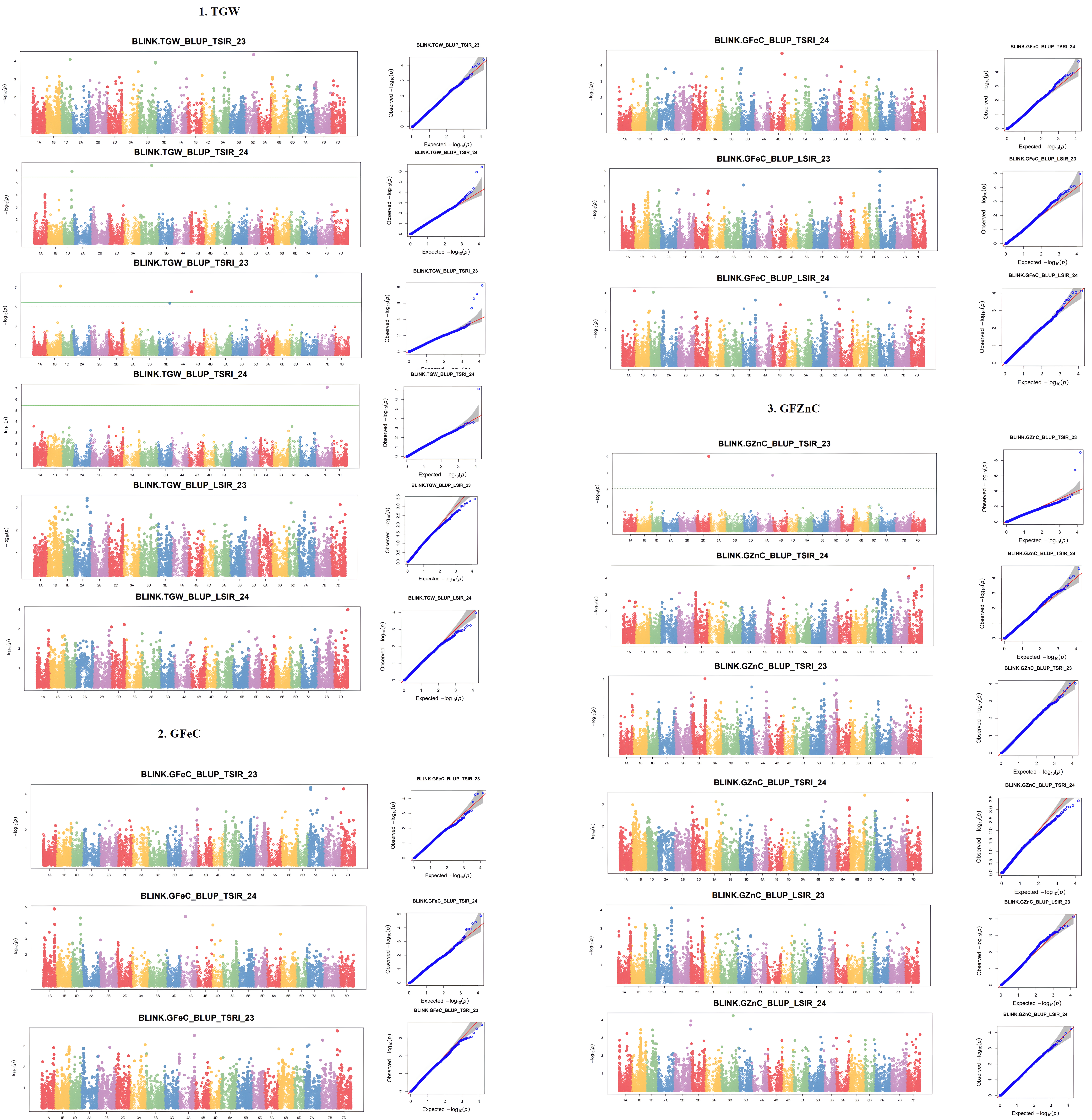

Supplement: S3 Fig — (TIFF) [file pone.0329578.s003.tiff]
