# Supplementary material for: Deciphering the genetic basis of grain iron and zinc content in wheat under heat and drought stress using GWAS
Source: PLoS One. 2025 Aug 14;20(8):e0329578. doi: 10.1371/journal.pone.0329578 (PMC12440253; doi:10.1371/journal.pone.0329578)
Supplement: Attachment — Submitted filename: Response to Reviewers_rebuttal letter.docx [file pone.0329578.s008.docx]

**Response to Reviewers**

We thank the Editor and Reviewers for their thoughtful and constructive feedback. Below, we provide detailed point-by-point responses. All changes made to the manuscript have been highlighted in the revised version using track change formatting.

**Response to the Academic Editor**

**Describing the candidate genes and SNP-derived allelic sequences through VCF files with VEP from using the ENSEMBL kind of database sequences for the SIFT score to find out the tolerant and deleterious mutations may enhance the value of the work. So in such a case, you can find out whether the SNP-derived mutations have the impact of the candidate gene-derived putative protein in the active site or any specific regions.**

Pfam and CATH are also good tools to study protein classification and structure. We have mentioned it in the discussion for detailed study of each proteins obtained to validate gene function and identifying functional alleles.

**Along with TGW, it could have been better if the grain yield had been used to compare effectively for correlations and marker trait associations.**

The present study focused on TGW as a yield component due to its higher heritability and lower environmental variability under stress. Grain yield, being a complex trait with strong genotype × environment interactions, poses greater challenges for precise genetic dissection across multi-environment trials.

**Typographical errors throughout the manuscript. Examples include Line 110, Line 58, Line 70, Line 151, Line 152, and Line 313.**

- Line 110: “The Iron and concentrations” → corrected to “The iron and zinc concentrations”
- Line 58: “Genetic bio-fortification” → corrected to “genetic biofortification”
- Line 70: Capitalization of “grain yield” → corrected
- Line 151: “Ensemble” → corrected to “Ensembl”
- Line 152 and Line 313: Removed extra spaces and standardized formatting
  All terminologies (e.g., BLUP, SNPs, GWAS) have been made consistent throughout the manuscript.
- Rewritten sentence as "The SNP AX-94689123 was located near the gene that encodes Wall-associated receptor kinase 2 protein."

**Comment 1:**

**Whether the study conducted at three environments (multi environmental based?) or study was conducted three conditions (irrigated, drought, heat stress) of the same environment, please	clarify?
Response:**
The study was conducted under three stress conditions (TSIR, TSRI, LSIR) at the same experimental location (ICAR–IARI, New Delhi) over two rabi seasons. We have revised the wording in the Abstract, Materials and Methods, and Discussion to clarify this this in the revised manuscript.

**Comment	2:**
**Do these 268 DNA samples, out of 280 genotypes, contain specific genotypes or varieties that are known for drought or heat tolerance? Have you used any check varieties for the traits used?
Response:**
In our study, we have included stress-tolerant genotypes such as HD3086 (heat-tolerant), HD3059 (drought-tolerant), and DBW187 (drought and heat-tolerant). Additionally, widely grown high-yielding cultivars such as HD3271 and HD3386 were used as check varieties for comparison. We have mentioned in the Materials and Methods section of revised manuscript.

**Comment 3:**

**Fig. 4A: PCA analysis may be done with different packages in R or other programs and may be produced effectively with principal components contributing the traits with the eigen vector's cosine of the angles and directions.**

**Response:**
The PCA was generated using GAPIT based on SNP data, while the dendrogram was based on a neighbor-joining tree. The discrepancy may be due to methodological differences between them, which is discussed in Discussion part. Future, GAPIT uses PCA as a covariate to avoid false association and is one of the recent approaches in GWAS. This PCA is based on genomic/marker data, hence trait vector is not drawn in the scatter plot.

**Comment 4:**

**The phylogenetic tree didn't show the bootstrap values. If it is possible, please add the bootstrap values at the nodes of the major clusters.**

**Response:**
We accept the value of including bootstrap values and will include them in upcoming studies. In current study we constructed the tree using TASSEL v5.0, which generates tree based on distance matrix. The tree is drawn using visualization tool, iTOL for better interpretation. To alien with the idea of this manuscript, we focused more on identifying marker trait associations.

**Comment 5:**

**Both GFeC and GZnC trait average values were higher for the late sown irrigated (LSIR); while the TGW was in an opposite trend with the lowest average at LSIR, it is looking like the mild heat stress with late sowing and normal irrigation may favor GFeC and GZnC accumulation over TGW. Have you compared any physiological observations and comparisons with GFeC and GZnC traits from literature on similar studies?
Response:**
We have explained and added references supporting stress-induced micronutrient accumulation due to the concentration effect and adaptive physiological responses (Velu et al., 2016; Alomari et al., 2018) in discussion part (L 354 -358 line number according to revised manuscript with track changes).

**Comment 6:**

**A strong negative correlation for TGW with GZnC and a weak positive correlation of the TGW with GFeC were observed with your study. I presume it is based on the phenotypic correlation, isn't it? Have you observed or referred to any other study about the genetic correlations among these traits besides phenotypic correlations?**

**Response:**
We used phenotypic correlations, however with BLUP values to reduce environmental influence on the traits. This is now clearly stated, and we added citations discussing genetic correlations among these traits (L 393-399)

**Comment7:**
**Explain faster LD decay in A sub-genome.** **Have you observed any of the specific paired genes (in LD) undergo LD decay, especially when comparing the linked genes from any of the chromosomes from the A sub-genome?**

**Response:**
In discussion we included the explanation for faster LD decay in the A sub-genome, may be due to higher recombination and the outcrossing nature of its progenitor species *Triticum urartu*. Previous studies have also, reported similar patterns, suggesting that the A genome usually exhibit more rapid this explanation is now added to the revised manuscript (L 422-426)

Pairwise LD between the markers (intra chromosomal) were computed before drawing the LD decay map. LD decay was drawn compared between the A, B and D genome. While we did not compute chromosome-specific LD decay curve, limiting it to sub-genome level.

**Comment 8:**

**Finding out about the MTA with 7A for GFeC and TGW and 7B for GZnC is interesting, having some consistency with a previous study as well. Similarly, you have also found other candidate genes from other chromosomes. However, the identified candidate genes can be described better through comparing their putative proteins with the CATH database, the Pfam database, etc.; it may provide additional clues related to biological functions and for comparing the related proteins, etc.**

**Response:**
Proteins and their biological functions of candidate genes were obtained from the Triticaceae-Gene Tribe website, wheat expression data base and interpro website. Major function of putative protein and related protein families were explored by these web and also from the previous studies published in various related crops. Pfam and CATH are also good tools to study protein classification and structure. We have mentioned it in the discussion for detailed study of each proteins obtained to validate gene function and identifying functional alleles.

**Comment 9:**

**Whether the superior genotypes identified are comparable with previously known cultivars that exhibit higher performance for these traits, especially for GZnC, since your study and other studies have revealed the negative correlations with yield-related parameters, in this case any superior genotypes/varieties are exempted from this negative correlation?**

**Response:**
Yes. Several of the eight top-performing genotypes (e.g., DBW332, RAJ4546) have GZnC levels (up to ~65 mg kg⁻¹ under stress) that rival or exceed those of biofortified varieties like ‘Zinc Shakti’ (~55–60 mg kg⁻¹).This point is now included in the Discussion (493-494).

**Editor Final Comment:**

**Improve figure resolution and revise the manuscript based on all suggestions**.

**Response:**
All figures have been updated for resolution and clarity. The manuscript has been revised in accordance with all reviewer and editor suggestions.

**Response to Reviewer #1**

**The aim of this work was to map genomic regions associated with grain zinc and iron contents and thousand-kernel weight under drought and heat conditions. The authors obtained a number of MTAs for three traits and found candidate genes. However, it is not clear which specific loci can affect the accumulation of microelements in grain under the influence of the studied abiotic stresses. And which genes start working under drought and heat compared to control conditions. The sections Abstract, Results, Discussion and Conclusion should be rewritten taking into account the stated goal (namely, the search for genomic regions under the influence of drought and heat)**

The Abstract(32-38), Results(259-273,279-293,295-308), Discussion(437-440) sections have been revised to align clearly with the study goal identifying genomic regions and genes influencing Fe, Zn, and TGW accumulation under drought and heat stress.

**Major Comments**

**1) The Introduction section is written in general phrases, which do not indicate the novelty of the study. The section should be expanded by describing in more detail the state of the work on QTL mapping (biparental mapping populations and GWAS) directly to the topic of the study - drought and high temperatures. The authors use the term abiotic stresses and claim that there are few studies on this topic, although at the moment there are quite a lot of published results on mapping to drought, salinity, high temperatures, moisture deficiency or excess, nutrient deficiency, etc. Information is also needed on the 1000-grain weight, since the authors study this trait further in the article, and on the relationship of the trait with the concentration of zinc and iron**

Introduction section is rewritten including all the suggestions. Current status of the work in GWAS and identification of QTL related to grain micronutrient content is described in length. Although, there are several studies on abiotic stress, studies with main focus on grain Zn and Fe content in relation to abiotic stress in wheat is few, they were well cited. Detail regarding TGW and micronutrient, their importance and aim of the study is rewritten in the introduction and discussion part for better understanding.

**2) The authors interpret phenotypic data for each year, while there are no results for the average values of traits and heritability for 2 years**

Average value of trait is depicted in the boxplots already and also separate table with all the details were given for trait values with heritability.

**3) It is necessary to add ANOVA data on the influence of genotype and environmental factors on phenotypic manifestation of the traits**

ANOVA tables showing genotype (G), environment (E), and G×E interaction effects have been added to the results. These clarify the environmental influence on trait expression under stress and non-stress conditions.

**4) I do not understand why the PCA analysis do not match the Structure data and the dendrogram? Use a different program for PCA visualization so that clustering is visible. What program was used to calculate the population structure shown in Fig. 4C?
PCA vs STRUCTURE vs NJ-tree differences** are discussed (408-416) in detail along with references

**5) Figure 6 is uninformative. Instead, a Manhattan plots should be added, constructed using average data (BLUP or BLUES) for 2 seasons and for all three traits**
The Manhattan plots based on BLUPs over two seasons under drought and heat conditions for all three traits included in main text (combined BLUPS; Fig 3) and supplementary (individual seasons ;S3_Fig).

**6) Discussion. Lines 261-271 are not a discussion, but resemble an introduction to the article.**
These lines have been removed and the Discussion now written to be analytical not introductory

**Minor comments:**

**1) The numbering of cited articles in the text is done chaotically and out of order. Sometimes citations are used not in digital form, but as Author et al.**

The references have been re-formatted in numerical order and in accordance with journal guidelines. Author-year citations have been converted to numbered references where appropriate.

**2) The methods do not provide a formula for calculating heritability in a narrow or broad sense**

We have now included the formula for broad-sense heritability in the Methods section (147-151)

**3) I recommend removing Figure 1 from the article, this is not a review, not a master's thesis, but an experimental article 4) Move lines 116-117 to section 2.3 5) Move Fig 2 to supplementary materials. 6) Move Figure 5 to supplementary materials.**

Figure 1 has been removed as suggested and Figures 2 and 5 moved to Supplementary. Line 116-117 to section 2.3

**What does the color scheme of the bars on the histogram mean?**

The histograms are standard ggplot2-style plots with bins automatically assigned different colors for visual distinction. The color scheme within each bar of the histograms appears to represent individual bins or ranges of phenotypic values within the dataset.

**7) Table 2 does not contain data on LSIR_CBLUP for zinc**

Table 2 had missing LSIR GZnC values because there was no significant MTAs (-log p value = 4) identified in LSIR_CBLUP

**Response to Reviewer #2**

**1. Introduction (language edits)**

All suggested language refinements have been addressed in the revised Introduction.

- “is the oldest” → changed to “is one of the oldest”
- “around approximately” → changed to “approximately”
- “poor micronutrient” → changed to “low micronutrient”
- “Bio-fortification” → changed to “Biofortification”
- “.The” → changed to “. The” (added space)
- “Genome-wide association studies (GWAS) (Fig.1)” corrected and abbreviation applied consistently

**2. Methods:**

**Inconsistent package reporting. Report the packages in “” and then in italic format and instead of R-studio mention e.g. “ggplot2” package in R. Instead of reporting the function mention the package name.**

All R packages are now cited in quotation marks and italicized. Package names (e.g., ggplot2) are used instead of individual functions.

**Remove “to the crop.”-** removed

**Inconsistent package reporting GAPIT package version136 3.041 and GAPIT version 3.0?!**
GAPIT version corrected and unified

**Why BLUP values instead of raw means? Justify using BLUP for controlling random effects or environmental noise, etc**

BLUP usage justified for environmental noise correction in methods section (176-179).

**3. Results:**

**You mention PCA showed no well-defined subpopulations, but phylogenetic clustering revealed eight subgroups. Please reconcile or discuss this discrepancy, why PCA might underrepresent this structure.**

This apparent discrepancy arises because PCA emphasizes the major axes of variation and may underrepresent finer hierarchical structures, while clustering methods can detect more subtle groupings based on overall genetic similarity, it has been added in discussion.

**Units inconsistency, genetic distance should be in cM or Mb consistently throughout the text. Clarify if this is physical or genetic distance.**

Fixed inconsistency in genetic/physical distances units for distance (cM/Mb) standardized.

**Explain why a 15% selection intensity was chosen, is this a standard practice or based on preliminary simulation outcomes?**

Explained 15% selection intensity based on standard MGIDI practices in results section (315-319). A 15% cut-off is commonly used in plant breeding to balance selection stringency and diversity retention.

**The phrase "higher contributing factors were located near the center" is ambiguous. Please clarify whether this refers to a graphical representation (e.g., factor analysis biplot) and how proximity to the center relates to contribution strength.**

We have clarified the interpretation of the MGIDI factor analysis biplot: traits farther from the center (origin) contribute more to genotype discrimination, while those closer to the center contribute less, as their loadings are smaller

**4. Discussion:**

**The broad genetic diversity is mentioned, but a quantitative diversity index (like PIC, Nei’s, or gene diversity) would solidify this claim**

While we did not perform marker-based diversity index calculations, we have revised the text to clarify that our interpretation is based on phenotypic variation revealed through ANOVA (344-346), which reflects the extent of variability within the panel relevant to our trait-based association analyses.

**"This might also be due to the concentration effect…", this important hypothesis would benefit from elaboration, perhaps with the reported supporting references on how stress-induced yield reduction increases grain micronutrient concentration.**

We elaborated on the "concentration effect" hypothesis, explaining how yield reduction under stress can increase grain micronutrient concentration. Supporting literature has been added (Lines 354–358).

**You correctly note that positive correlation suggests shared mechanisms. It would strengthen the discussion to propose possible physiological pathways or QTL co-localization evidence with supporting references.**

We have included possible physiological explanations and cited studies on co-transport and QTL co-localization to support the observed positive correlation between Fe and Zn (Lines 381–390).

**The discrepancy between PCA and phylogenetic tree clustering (as noted earlier) needs a deeper discussion on potential causes like low within-cluster variance or marker informativeness.**

PCA/tree inconsistency is discussed in more depth i.e. due to low within-group variance and differences in clustering methods and marker informativeness (Lines 408–419).

**The discussion around candidate genes is sound, but most gene functions are general stress-related. It would add depth to mention whether these genes were previously implicated in nutrient homeostasis, specifically Fe and Zn.**

Candidate gene discussion expanded with references on genes involved in Zn and Fe transport and homeostasis, not just general stress responses (440-443,457-460,479-484).

**Excellent identification of repeatedly implicated regions. Suggest adding a sentence proposing future fine-mapping or functional validation in these hotspot regions (like 7A) to solidify these associations.**

Suggestions for future fine-mapping have been conclusion
