# Supplementary material for: Deciphering the genetic basis of grain iron and zinc content in wheat under heat and drought stress using GWAS
Source: PLoS One. 2025 Aug 14;20(8):e0329578. doi: 10.1371/journal.pone.0329578 (PMC12440253; doi:10.1371/journal.pone.0329578)
Supplement: Attachment — Submitted filename: Response_to_Reviewers_Rebuttal_letter_auresp_2.docx [file pone.0329578.s009.docx]

**Rebuttal Letter**

Manuscript Title: **Deciphering the Genetic Basis of Grain Iron and Zinc Content in Wheat under Heat and Drought Stress Using GWAS**

Dear Editor and Reviewer,

We thank you for your detailed feedback, we have carefully addressed all the comments raised and made the required revisions.

As per the journal requirements, we have carefully checked our reference list. We confirm that none of the references we have cited have been retracted. All references are complete with correct author names, years, journal names, volume, pages, and DOI links where needed.

**Editor Comments:**

**1️.”Please check for typos and spacing in line 156.”**
Corrected a minor spacing inconsistency identified in the sentence for clarity.

**2. “Please correctly mention existing conventions for Neighbour-joining (NJ) and change intermittent rains to intermittent rain.”**
 We have corrected “Neighbour-joining” to “Neighbor-Joining (NJ)” throughout the manuscript with standard conventions.
 We have replaced “intermittent rains” with “intermittent rain” as suggested.

**3️. “It is better to indicate the months falling in the rabi season in brackets; please check line 108.”**
The months for the *rabi* season (November to March) are now specified in brackets in line 108 for clarity.

**4️. “Please indicate which version of MEGA.”**
 Included “MEGA X version 10.2.6” in the Methods section (Line: 398) where MEGA is mentioned.

**5. “Please italicize the genus Arabidopsis in line 446.”**
The genus *Arabidopsis* has been italicized in line 446.

**6️. “It is better to provide the importance of the candidate genes and mention specific candidate genes with high scores for Zn and Fe in conclusion.”**
 We have added the following sentence in the Conclusion:

“Notably, SNP AX-95001849 (*TraesCS2D02G503000*, plasma membrane ATPase) for GZnC and SNP AX-94953068 (*TraesCS7A02G171600*, galactoside 2-alpha-L-fucosyltransferase) for GFeC under stress emerged as key candidates. These loci underscore potential targets for biofortification breeding to enhance grain Zn and Fe content under heat and drought stress.”

**7️. “Check for typos, space errors, etc.”** **I suggest thoroughly revising the manuscript for these minor errors.**
 We have thoroughly rechecked the entire manuscript to correct minor typos, inconsistent spacing, and formatting issues.

**Reviewer #1 Comment:**

1. **“What program was used to calculate the population structure shown in Fig. 4C? If STRUCTURE was used, indicate in Methods with reference.”**
   We confirm that STRUCTURE software version 2.3.4 (Pritchard et al., 2000) was used and the results were visualized using iTOL version 6.5.2. it has been updated the Methods section (Line:159-161) accordingly and included the following reference:

Pritchard JK, Stephens M, Donnelly P. Inference of population structure using multilocus genotype data. Genetics. 2000;155 (2):945–959. <https://doi.org/10.1093/genetics/155.2.945>

2. **“Corrected bio-fortification to biofortification (line 39, 64, 65, 484).”**
   Corrected the“bio-fortification” to “biofortification” in all indicated lines.
3. **“Corrected Insilco to in silico (line 161).”**
   We have corrected “Insilco” to “in silico” in line 161.
4. **“Table 3: Specify units in the column Position – Mb or bp.”**
    The unit “Mb” in the “Position” has been specified column header in Table 3 for clarity.
5. **“Table 3: MTAs detected multiple times are highlighted in bold text. Forgot to mark in bold??”**
    MTAs detected multiple times now are highlighted in **bold text** in Table 3 as intended.
6. **“Table 3: Corrected P.value to p-value.”**
    We have corrected “P.value” to “p-value” in Table 3.

We thank you once again for your time and consideration and look forward to your positive response.
